# *Notes from the Field*: Public Health Response to Surveillance for Recent HIV Infections — Malawi, May 2024

**DOI:** 10.15585/mmwr.mm7420a4

**Published:** 2025-06-05

**Authors:** Reno Stephens, Harriet Mfungwe, Davie Chalira, Misheck Luhanga, Joe Theu, Romance Thawi, Kelly Chapman, Victor Singano, James Jere, Christopher Blair, Gabrielle O’Malley, Monita Patel, Alex Ernst, Rashida Hassan, Alinune Kabaghe, Melissa M. Arons

**Affiliations:** ^1^Division of Global HIV & TB, Center for Global Health, CDC Malawi; ^2^Public Health Institute, Oakland, California; ^3^International Training and Education Center for Health, Department of Global Health, University of Washington, Lilongwe, Malawi; ^4^Malawi AIDS Counselling and Resource Organization, Lilongwe, Malawi; ^5^Division of Global HIV & TB, Center for Global Health, CDC; ^6^Public Health Institute of Malawi, Lilongwe, Malawi; ^7^International Training and Education Center for Health, Department of Global Health, University of Washington, Seattle, Washington; ^8^Global Strategic Information, Institute for Global Health Sciences, University of California, San Francisco, California.

**Summary**

**What is already known about this topic?**

Surveillance for recent HIV infections (i.e., recent HIV infection surveillance) classifies newly diagnosed infections as recent (acquired during the past 12 months) or long-term (acquired >12 months ago), allowing for potential identification of geographic areas with ongoing acquisition.

**What is added by this report?**

In Malawi, spatial analysis of surveillance data identified eight clusters of facilities with higher-than-expected recent HIV infections, prompting a facility-level public health evaluation and response. The public health response team used program data and semistructured interviews with community representatives to identify and address service delivery gaps, which included low viral load suppression levels among persons receiving HIV antiretroviral treatment and low rates of prescribing preexposure prophylaxis.

**What are the implications for public health practice?**

Combined with other data sources, recent HIV infection surveillance can identify opportunities for interest holder engagement and targeted interventions to enhance HIV programming and contribute to HIV/AIDS epidemic control.

Despite Malawi’s progress toward HIV epidemic control, an estimated 12,000 new HIV infections occurred in the country in 2023.* Surveillance for recent HIV infections (recent HIV infection surveillance) involves use of a recent infection testing algorithm that combines the result of a rapid test for recent infection with a viral load result to classify newly diagnosed HIV infections as recent (likely acquired during the past 12 months) or long-term (acquired >12 months ago) (*1*). Recent HIV infection surveillance enables detection of geographic areas associated with potential ongoing HIV acquisition (*2**,**3*). This report describes the public health response to a signal (i.e., the detection of a rate of recent HIV infection that is higher than expected) at a public health clinic in Malawi’s southern region in May 2024.

## Investigation and Outcomes

As part of routine recent HIV infection surveillance in Malawi, data collected during April–September 2023 were analyzed using spatial scan statistics, a method for detecting signals (clusters of health care facilities with rates of recent HIV infections that are statistically significantly higher than those expected due to random variation) (*3*). Rates of recent infection were calculated as the number of recent HIV infections per 100,000 persons at risk for HIV.^†^ Among 289 facilities implementing recent HIV infection surveillance, 26 facilities were identified through eight signals with rates of recent HIV infections that were higher than expected; public health responses were initiated at all 26 facilities. This report details the response at one of two public health facilities in Malawi’s southern region within a signal (expected recent HIV infection rate = 3.36 per 100,000; actual recent HIV infection rate = 16 per 100,000). These activities were reviewed by CDC, deemed not research, and were conducted consistent with applicable federal law and CDC policy.^§^

After verifying facility-level HIV testing and diagnosis data using nationally reported data, a multidisciplinary public health team, including governmental and nongovernmental interest holders and facility health care workers (HCWs), initiated response activities. Data were reviewed from seven predetermined program indicators included in the U.S. President’s Emergency Plan for AIDS Relief Monitoring Evaluation and Reporting Database^¶^ (covering testing, prevention, and treatment programs during April 2022–September 2023) alongside site-level data to identify gaps in HIV service delivery.** Two focus groups including 19 community representatives used a semistructured interview guide to discuss the identified gaps. Community representatives included faith-based community leaders, shop owners, and leaders from HIV community groups. The program indicators, identified service gaps, and interview findings were summarized and presented to the multidisciplinary public health team, which developed recommendations and action plans to address the identified service gaps.

One service gap identified at the public health facility was that levels of viral load suppression^††^ among persons receiving antiretroviral therapy (ART) were consistently lower than the established target of ≥95%. This issue was particularly apparent among adolescents and young adults aged 15–19 years, whose viral load suppression range was 53%–59%. ART adherence is central to achieving viral load suppression. Although adherence to ART was not measured, barriers identified through qualitative interviews with interest holders included parental hesitancy to disclose HIV status to adolescents and young adults who had acquired HIV infection perinatally (*4**,**5*), inaccurate care and treatment information from faith-based community leaders, and a lack of privacy during ART adherence counseling.^§§^

A second identified service gap was stagnation at low levels in the number of preexposure prophylaxis (PrEP) prescriptions despite a general increase in availability of PrEP during the preceding 18 months. The number of persons newly initiated on PrEP fluctuated from 13 to 36 clients with no increase during the 18-month period. Semistructured interviews revealed that slow adoption of PrEP use might be attributed to HIV-related stigma, low awareness of PrEP availability in the community, and insufficient numbers of HCWs trained in PrEP provision. PrEP services had been relocated away from ART services in early 2023, in an effort to address HIV-associated stigma; however, this action and the continued availability of PrEP were not well communicated to the community.

## Preliminary Conclusions and Actions

To increase levels of viral load suppression, the response team recommended enhanced adherence counseling,^¶¶^ educating and involving faith-based community organization leaders in ART advocacy, and promoting HIV disclosure sessions among parents and adolescents. To improve adoption of PrEP, interest holders agreed to train more HCWs as PrEP providers, integrate PrEP provision at multiple service points, and conduct community awareness campaigns. This application of a public health response to recent HIV infection surveillance highlights one way to use timely surveillance data to identify service delivery gaps and contribute toward a goal of controlling the HIV/AIDS epidemic.

## Footnotes

† The number of recent HIV infections divided by the sum of the number of recent infections plus the total number of negative HIV test results multiplied by 100,000.

§ 45 C.F.R. part 46.102(l)(2), 21 C.F.R. part 56; 42 U.S.C. Sect. 241(d); 5 U.S.C. Sect. 552a; 44 U.S.C. Sect. 3501 et seq.

¶ Site-level monitoring evaluation and reporting indicators reviewed for the previous 18 months included 1) the number of persons who received HIV testing services and received their test results; 2) the number of persons aged ≥15 years who received HIV testing services and received a positive test result; 3) the number of persons who were identified and received testing using Index Testing Services (a voluntary service by which HCWs assist persons who receive a positive HIV test result with identifying sexual partners and biological children and adolescents and young adults aged <19 years at risk for HIV infection) and received their test results; 4) the number of persons newly enrolled for antiretroviral therapy (ART); 5) the number of persons receiving ART; 6) the percentage of persons receiving ART and who had a suppressed viral load result; and 7) the number of persons who were newly enrolled to receive preexposure prophylaxis to prevent HIV infection.

**
Monitoring, Evaluation, and Reporting Indicator Reference Guide – U.S. President’s Emergency Plan for AIDS Relief

†† A viral load test result of <1,000 copies of HIV per mL of blood.

§§
Consolidated Guidelines on HIV Prevention, Testing, Treatment, Service Delivery and Monitoring: Recommendations for a Public Health Approach – World Health Organization

¶¶ Enhanced adherence counseling includes a baseline individual needs assessment, adherence counseling and education sessions, and follow-up telephone calls.

## References

[1] Yufenyuy EL, Detorio M, Dobbs T, Correction: performance evaluation of the Asante Rapid Recency Assay for verification of HIV diagnosis and detection of recent HIV-1 infections: implications for epidemic control. PLOS Global Public Health 2023;3:e0002287. 10.1371/journal.pgph.0002287 37540640 PMC10403083

[2] Kim AA, Behel S, Northbrook S, Parekh BS. Tracking with recency assays to control the epidemic: real-time HIV surveillance and public health response. AIDS 2019;33:1527–9. 10.1097/QAD.0000000000002239 31021850 PMC6776479

[3] Telford CT, Tessema Z, Msukwa M, Geospatial transmission hotspots of recent HIV infection—Malawi, October 2019–March 2020. MMWR Morb Mortal Wkly Rep 2022;71:329–34. 10.15585/mmwr.mm7109a1 35239633 PMC8893337

[4] Mengesha MM, Teshome A, Ajema D, Tura AK, Hallström IK, Jerene D. The association between HIV diagnosis disclosure and adherence to anti-retroviral therapy among adolescents living with HIV in Sub-Saharan Africa: a systematic review and meta-analysis. PLoS One 2023;18:e0185571. 10.1371/journal.pone.0285571 37167342 PMC10174542

[5] Cluver LD, Hodes RJ, Toska E, ‘HIV is alike a tsotsi. ARVs are your guns’: associations between HIV disclosure and adherence to antiretroviral treatment among adolescents in South Africa. AIDS 2015;29:S57–65. 10.1097/QAD.0000000000000695 26049539

